# Olive Fruit Ripening Degree and Water Content Relationships with Phenolic Acids and Alcohols, Secoiridoids, Flavonoids and Pigments in Fruit and Oil

**DOI:** 10.3390/molecules28196943

**Published:** 2023-10-05

**Authors:** Giulia Vicario, Claudio Cantini, Alessandra Francini, Andrea Raffaelli, Mario Cifelli, Valentina Domenici, Luca Sebastiani

**Affiliations:** 1 Crop Science Research Centre (CSRC), Scuola Superiore Sant’Anna, Piazza Martiri della Libertà 33, 56127 Pisa, Italy; giulia.vicario94@gmail.com (G.V.); a.francini@santannapisa.it (A.F.); andrea1.raffaelli@santannapisa.it (A.R.); 2 Institute for BioEconomy (IBE), National Research Council of Italy (CNR), Via Vecchia Aurelia 49, 58022 Follonica, Italy; claudio.cantini@ibe.cnr.it; 3 Institute of Agricultural Biology and Biotechnology—National Research Council (IBBA—CNR), Via Moruzzi 1, 56124 Pisa, Italy; 4 Chemistry and Industrial Chemistry Department, University of Pisa, Via Moruzzi 13, 56124 Pisa, Italy; mario.cifelli@unipi.it (M.C.); valentina.domenici@unipi.it (V.D.)

**Keywords:** olive fruit, phenols, pigments, olive oil, maturity index

## Abstract

Olive drupe traits (i.e., ripening index and pericarp water content) and minor components (i.e., phenols and pigments in both fruit and oil) are important for human health and are affected by agronomic background. The aim of this study was to investigate the relationship between fruit traits, phenols, and pigments in samples derived from different soil and water management practices. Chromatographic (UHPLC-MS/MS) and spectroscopic (^1^HNMR and near UV-Vis spectroscopy) techniques were employed for the characterization of olive fruits and oils. The use of various techniques allowed the identification of interesting trace compounds. We observed that most of the fruit phenols (a total of 29 compounds) were correlated with the degree of ripening: most of the phenolic acids (and their derivatives), phenolic alcohols, and secoiridoids were negatively correlated, whereas the majority of the studied flavonoids were positively correlated. The relationship between the ripening index and fruit phenolic compounds appears to be dependent on the metabolic pathway that controls the synthesis of each individual compound. Conversely, the secoiridoids and pigments in olive oil showed a negative correlation with pulp moisture, probably because of the influence of the water content on the extractability and transfer in the oil phase of these minor components.

## 1. Introduction

Olive products are known for their unique sensory and nutritional attributes. Most of these characteristics depend on the composition of the minor fraction, which includes phenols and pigments, among others [1,2].

Hydrophilic phenols range from 1 to 3% in olive pericarp (fresh weight), and various phenol subgroups can be found (phenolic acids, phenolic alcohols, lignans, flavonoids, and secoiridoids) [3]. Mechanical extraction allows the transfer of phenols to the oil fraction. During this process, changes in the chemical structures and relative abundance of individual compounds occur, and specific fruit enzymes (β-glucosidase, polyphenol oxidase, and peroxidase) participate in this process [4,5]. One of the main targets of these enzymes is secoiridoids, which are typical of the Oleaceae family and strongly contribute to the organoleptic (bitter and pungent attributes) and antioxidant properties [6]. Among the secoiridoids, oleuropein and ligstroside are glycosylated compounds mainly found in the fruit, whereas 3,4-DHPEA-EDA (oleacein) and p-HPEA-EDA (oleocanthal), as well as the aglycone forms of both oleuropein and ligstroside, are non-glycosylated secoiridoids mainly reported in olive oil. Similar to secoiridoids, phenolic alcohols, such as tyrosol (p-HPEA) and hydroxytyrosol (3,4-DHPEA) and their derivatives, play a relevant role in the nutritional properties of olive oil, as also reported by an EFSA health claim [7]. They are structurally simpler compared to secoiridoids, and they are present in both olive fruits and oils. The less abundant phenolic fractions include phenolic acids, lignans, and flavonoids. Methoxylation and hydroxylation of the aromatic ring of hydroxybenzoic and hydroxycinnamic carbon skeletons generate a large number of the phenolic acids present in olive fruit and oil, which can be further modified to generate important derivatives (i.e., chlorogenic acid and verbascoside) [3]. Flavonoids in olive products mainly include anthocyanins, luteolin, apigenin, quercetin, and their glycosidic derivatives, of which pinoresinol and its derivatives are the most relevant [3,8].

Pigments are responsible for the color of olive oil and for some of its beneficial health properties. Chlorophylls (mainly chlorophyll *a* and *b*) and carotenoids (mainly lutein and β-carotene) are present in the olive pericarp and are involved in photosynthesis [9]. Pigments are transferred from fruit to oil, but significant changes occur depending on many factors, such as light and oxygen exposure [10]. Chlorophylls (*a* and *b*) are converted to pheophytins (*a* and *b*, respectively), which are still colored compounds due to the typical acidity of oil and, later, depending on oil storage conditions, they can transform into uncolored pyropheophytins. However, carotenoid degradation during oil extraction and storage leads to uncolored products [11]. Generally, pheophytin *a*, among chlorophylls, and lutein and β-carotene, among carotenoids, are the most abundant pigments in olive oil [12].

The crucial role of genotype, harvest time in terms of ripening index, environmental factors (pedoclimatic conditions and geographical origin), soil and water management, biotic stresses [13], extraction (crushing and malaxation), and storage conditions have been demonstrated to strongly influence the abundance of phenols and pigments. During ripening, phenolic and pigment reductions are genotype- and single-compound dependent [14,15,16,17]. For instance, comparing green vs. black stage fruits, oleuropein is more drastically reduced in cv Arbequina (−97%) compared to cv Cornicabra (−55%) [18]. Comparing oils (cv Arbequina) derived from unripe and ripe fruits, the same study revealed that 3,4-DHPEA-EDA was reduced more (−39%) than p-HPEA-EDA (−32%). Chlorophylls are degraded more rapidly than carotenoids in Arbequina, and the degradation of the carotenoid fraction occurs only at complete ripeness [19].

Ripening is genetically regulated, but water and soil management practices largely influence the maturation process, as well as other fruit traits (i.e., water content in the pericarp). For example, irrigation induces consistent changes in ripening dynamics, fruit oil, and water content [20], and the use of various types of groundcovers can affect ripening and quality traits of the fruit and oil [21]. Moreover, irrigation can shape the phenol profile of olive fruit, thereby influencing enzyme activities (especially L-Phenylalanine ammonia-lyase) [22,23,24].

The aim of this study was to investigate the relationship between minor compounds, such as phenols (phenolic acids and alcohols, secoiridoids, flavonoids, and lignans) and pigments and specific fruit traits (ripening index and water content). To obtain the greatest variability in traits, fruit samples were obtained from orchards grown under different agronomic backgrounds for soil and water management. In addition, various analytical techniques (UHPLC-MS/MS, ^1^H NMR, and near UV-Vis spectroscopy) were employed. and trace/unidentified compounds were identified. The cultivar Arbequina was selected because it has been well studied and widely adopted in olive cultivation worldwide [25].

## 2. Results

### 2.1. Fruit Traits

**Figure 1:**
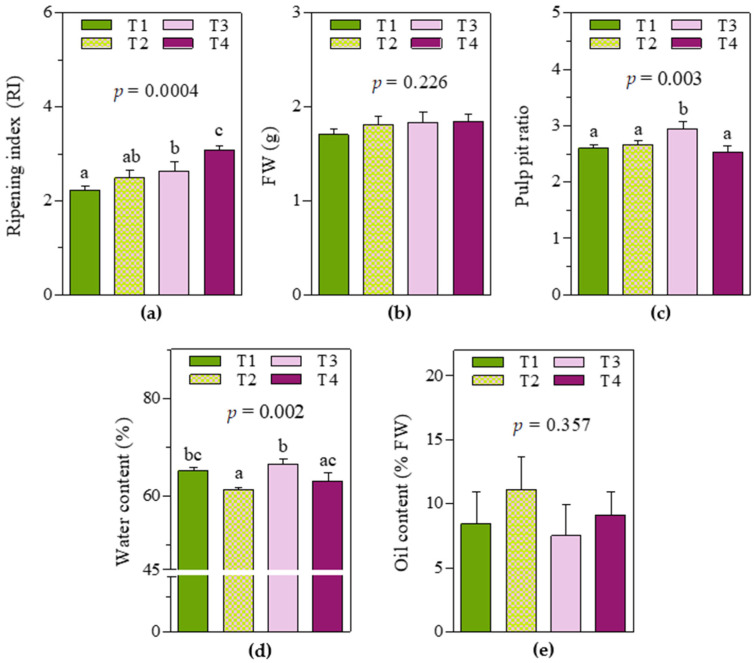
Mean values ± standard deviations of fruit traits: (**a**) ripening index; (**b**) fresh weight (FW) of a single fruit (g); (**c**) pulp–pit ratio; (**d**) water content in the pericarp (%); (**e**) oil content in the pericarp (% fresh weight, FW) of fruit samples from olive (‘cv Arbequina’) trees subjected to T1, T2, T3, and T4. Data were analyzed using one-way ANOVA Tukey’s post hoc test at a probability level of 0.05, and different letters indicate significant differences. *p*-values are reported in graphs.

As expected, soil and water management practices determined consistent variability in fruit characteristics at harvest time (Figure 1). Significant differences were reported, especially in the ripening index and water content in the pericarp, whereas no significant effect was observed in the fresh weight of a single whole fruit or pericarp oil content. In detail, T3 and T4 fruits showed the highest ripening indexes (2.6 ± 0.15 and 3.1 ± 0.06, respectively), while in T1 fruits, the ripening index was significantly lower (2.2 ± 0.08, −15% and −29% respect to T3 and T4 fruits, respectively). For T2 fruits, an intermediate value (between that of T1 and T3 fruits) was observed (2.5 ± 0.14). Regarding water content (%), T3 fruits showed the highest value (66 ± 0.9), whereas T2 fruits showed the lowest (61 ± 0.2). Moreover, T1 and T4 fruits showed similar intermediate values but were significantly different from T2 and T3, respectively. It is worth noting that even if no significant differences were observed between T2 and T1/T3 fruits in terms of ripening index, they were significantly different in terms of water content. Regarding the pulp–pit ratio, T3 fruits showed the highest value (2.9 ± 0.09).

### 2.2. Correlation Analysis

**Figure 2:**
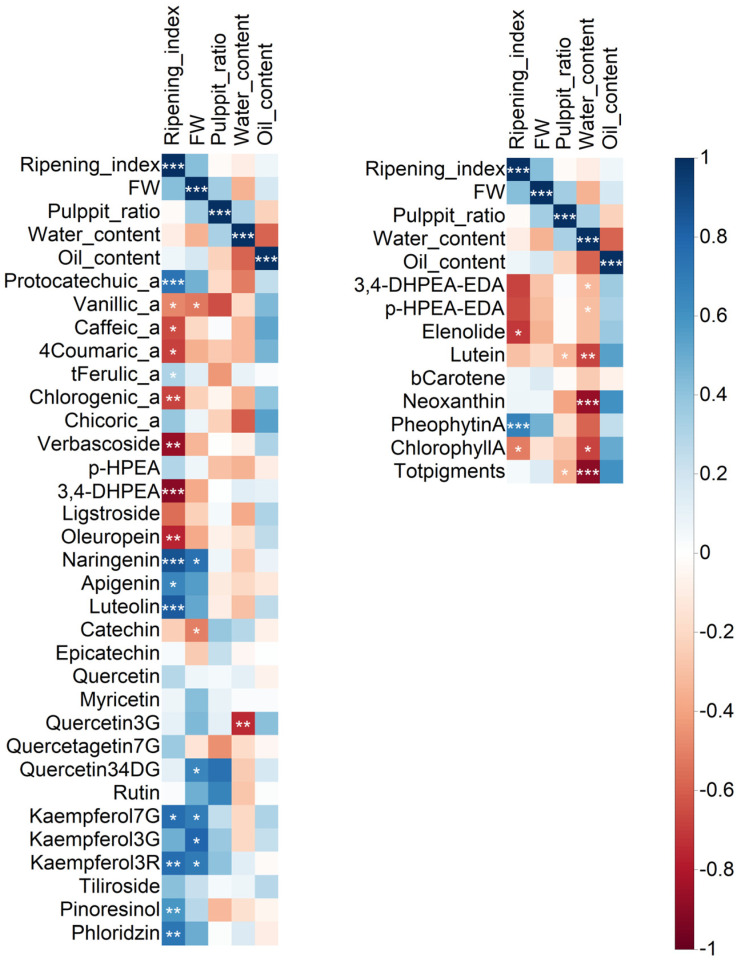
Correlation matrix based on Spearman’s correlation coefficients among fruit pomological traits, fruit phenolic compounds (left), secoiridoids (and derivatives), and pigments in oil (right). The following abbreviations were used: a = acid, G = glucoside, DG = diglucoside, R = rutinoside, and b = beta. Color intensity was proportional to Spearman’s coefficients. Positive correlations are shown in blue, and negative correlations are shown in red. The significance of Spearman’s coefficients is indicated by asterisks: * = *p* ≤ 0.01, ** = *p* < 0.001, *** = *p* < 0.0001.

As a considerable amount of data regarding fruits and oils were obtained, a correlation analysis was performed to determine the relationships between the variability in fruit traits and the phenol and pigment data (Figure 2). In addition, this analysis allowed us to identify the most closely related parameters, those significant at *p* ≤ 0.05, and select them for further discussion. The entire numeric matrix (correlation coefficients) and *p*-values associated with the correlation coefficients are reported in the Supplementary Material (Tables S1 and S2, respectively).

A principal component analysis (PCA) was carried out with the aim of studying olive fruit ripening degree and water content relationships with phenolic acids and alcohols, secoiridoids, flavonoids and pigments in fruit and oil (Figures S1–S4). The first two axes of the PCA of phenolic acids and derivate explained a total of 81.8% of the variability between the variables (Figure S1). As concern phenolic alcohols, secoiridoids and derivates, the first two axes of the PCA explained a total of 90.7% of the (Figure S2). Finally, the first two axes of the PCA of flavonoids and lignin (Figure S3) and pigments (Figure S4) explained a total of 75.9% and 93.8% of the variability, respectively.

For fruit analysis, 41 phenolic compounds were analyzed using UHPLC-MS/MS. Additionally, 29 compounds were identified and quantified in the pericarp. Apart from those usually present in olive fruits, some trace compounds not currently reported in the literature (catechin, epicatechin, and quercetin) or, to the best of our knowledge, have not been described in olive fruits (chicoric acid, myricetin, kaempferol-3G, kaempferol-3R, quercetagetin-7G, quercetin-3,4-DG, tiliroside, and phloridzin) were likely found. Some phenolic compounds were abundant but not usually reported (i.e., protocatechuic acid, chlorogenic acid, quercetin-3G) or not identified (i.e., kaempferol-7G). Interestingly, a wide group of compounds (protocatechuic acid, t-ferulic acid, naringenin, apigenin, luteolin, kaempferol-7G, kaempferol-3R, phloridzin, and pinoresinol), most of which are flavonoids, were positively correlated with the ripening index, while some other compounds (vanillic acid, caffeic acid, 4-coumaric acid, chlorogenic acid, verbascoside, 3,4-DHPEA, ligstroside, oleuropein), mostly phenolic acids and derivates, phenolic alcohols, and secoiridoids, were negatively correlated with the degree of ripening. Quercetin-3G showed a significant negative correlation with pericarp water content.

Regarding olive oils, secoiridoids and their derivatives and pigments were analyzed using spectroscopic techniques (^1^H NMR and near UV-Vis spectroscopy, respectively). Both 3,4-DHPEA-EDA and p-HPEA-EDA appeared to be negatively correlated with water content. ^1^H NMR analysis revealed the presence of S-(E)-elenolide, a non-phenolic compound putatively derived from fruit secoiridoids (oleuropein and ligstroside) [26]. Correlation analysis revealed a significant negative correlation between S-(E)-elenolide and ripening index. Most pigments (lutein, 9-cis neoxanthin, chlorophyll *a*, and total pigments) were negatively correlated with water content. Conversely, pheophytin *a* was positively correlated with the degree of ripening, differing from chlorophyll *a,* which was negatively correlated.

The phenolic compounds/pigments that showed significant differences among treatments and/or significant correlations with the ripening index and/or pericarp water content will be described in detail in the further subsections, while the numerical data for the other compounds are reported in Table S3.

In further subsections, phenols are described according to their subgroups (phenolic acids and derivatives, phenolic alcohols and secoiridoids, flavonoids, and lignan), and pigments are presented.

### 2.3. Phenolic Acids and Derivates

**Figure 3:**
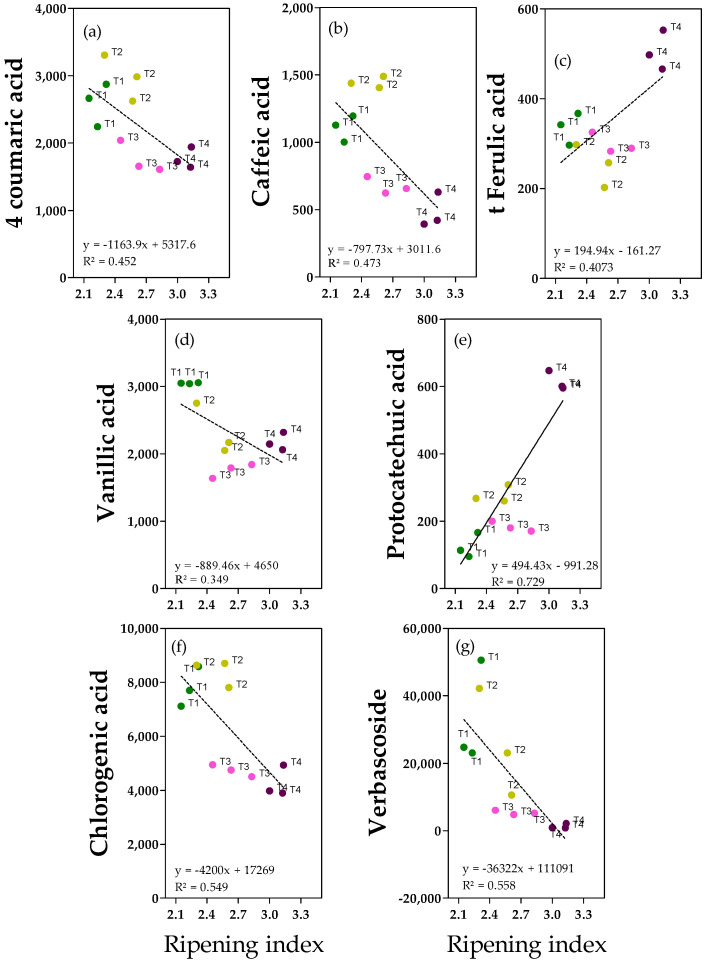
Regression analyses between ripening index and phenolic acids and derivates (ng g^−1^ FW): (**a**) 4-coumaric acid; (**b**) Caffeic acid; (**c**) *trans*-ferulic acid (t-ferulic acid); (**d**) Vanillic acid; (**e**) Protocatechiuc acid; (**f**) Chlorogenic acid; (**g**) Verbascoside in fruits from olive (‘cv Arbequina’) trees subjected to T1 (•) full irrigation with tilled soil, T2 (•) reduced irrigation with tilled soil, T3 (•) full irrigation with non-tilled soil and, T4 (•) reduced irrigation with non-tilled soil.

Phenolic acids were less abundant in fruits and oils. Data show that there were clear linear relationships between the ripening index and the variables 4-coumaric acid, caffeic acid and verbascoside in olive fruits (Figure 3a,b,g). In other cases, there is not a homogeneous and linear dispersion of samples along the ripening index values (Figure 3c–f), making these relationships weak. The 4-coumaric and vanillic acids were the most relevant (1.6 and 3.3 mg kg^−1^) (Figure 3a,d and Figure S5). For 4-coumaric acid, significantly higher values were detected in the lower fruit ripening index compared to the higher ripening index values (−37% on average in more ripe fruits), in accordance with the negative correlation with ripening degree observed in the correlation analysis (Figure 3a). Interestingly, for vanillic acid, the highest value was reported in more unripe fruits and the lowest in T3 fruits, with −41% in ripe fruits (Figure S5). Caffeic acid was less abundant but similar to 4-coumaric acid, the lowest amount was reported in fruits with a higher ripening index, as predicted through the use of correlation analysis (Figure 3b). Considering T2 fruits (which reported the highest caffeic acid concentration), an average reduction of −61% was observed in T3-T4 fruits (Figure S5). Different behaviors were observed for t-ferulic acid and protocatechuic acid (Figure 3c and Figure 3d, respectively). For both compounds, the highest concentrations were reported in fruits with a higher ripening index; in particular, t-ferulic acid showed +42% in ripe fruits (T4) compared to the average amount in the other fruits, while protocatechuic acid showed +74% in T4 ripe fruits compared to the average amount in T1 and T3 fruits (Figure S5). T2 fruits had intermediate values. Chlorogenic acid and verbascoside (Figure 3f and Figure 3g, respectively) are caffeic acid derivatives present mainly in olive fruit. Chlorogenic acid was found in our fruits at relatively high concentrations (ranging from 4 to 8 mg kg^−1^) and was significantly higher in less ripe fruits (T1 and T2), similar to 4-coumaric acid (−44% in T3 and T4 fruits) (Figure S5). Verbascoside was more abundant than phenolic acids (0.8–50 mg kg^−1^), and fruits with a higher ripening index showed a significantly lower amount compared to less ripe fruits (−96%) (Figure S5).

### 2.4. Phenolic Alcohols, Secoiridoids and Derivates

**Figure 4:**
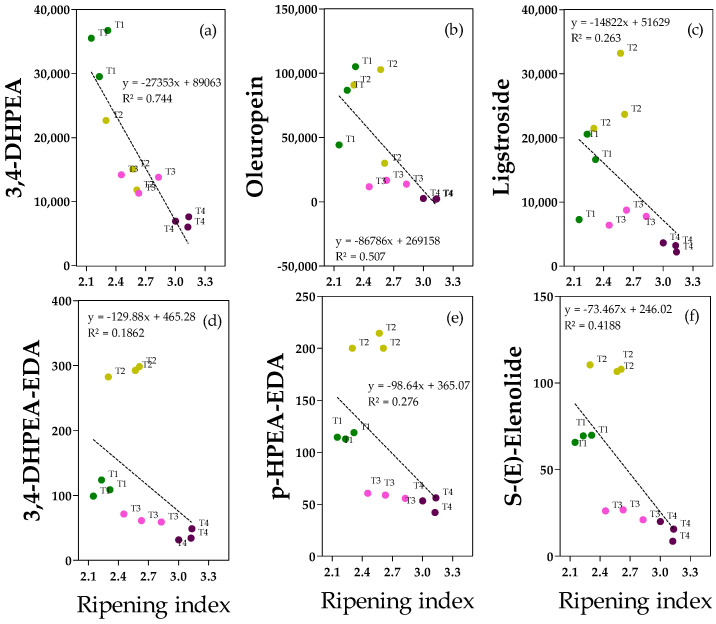
Regression between ripening index and phenolic alcohols, secoiridoids, and derivatives (mg kg^−1^ FW): (**a**) 3,4-DHPEA (hydroxytyrosol); (**b**) oleuropein; (**c**) stroside; (**d**) 3,4-DHPEA-EDA (oleacein); (**e**) p-HPEA-EDA (oleocanthal); (**f**) S-(E)-elenolide in fruits (**a**–**c**) or in oils (**d**–**f**) from olive (‘cv Arbequina’) trees subjected to T1 (•) full irrigation with tilled soil, T2 (•) reduced irrigation with tilled soil, T3 (•) full irrigation with non-tilled soil and, T4 (•) reduced irrigation with non-tilled soil.

Concerning phenolic acid and secoiridoids, the data show that there were clear linear relationships between the ripening index and the variables 3,4-DHPEA (hydroxytyrosol, Figure 4a) in olive fruits. Hydroxytyrosol (Figure 4a) is one of the most important antioxidant compounds found in olive fruit and oil. Its concentration in the fruit sample (6–37 mg kg^−1^) was negatively correlated with the ripening degree, and ripe fruits (T4) were significantly lower than those of T1 and T2 fruits (−80% and −58%, respectively) (Figure S6). Similar to 3,4-DHPEA, secoiridoids (oleureopein and ligstroside, Figure 4b,c, respectively) in olive fruits were negatively correlated with the ripening index, but in this case, there is not a homogeneous and linear dispersion of samples, and significantly lower values were reported in ripe fruits than in less ripe fruits (T1 or T2). Oleuropein was abundant, especially in T1 and T2 fruits (ranging from 30 to 105 mg kg^−1^), whereas it was drastically reduced in ripe fruits (−97% compared to the average of T1 and T2 fruits) (Figure S6). Regarding ligstroside, it was less abundant compared to oleuropein (the highest values were reached in T2 fruits, 26 ± 5.1 mg kg^−1^), but analogously to oleuropein, it was drastically reduced in ripe fruits (−71% and −88%, respectively, compared to T2) (Figure S6).

Concerning secoiridoids in olive oils, ^1^H NMR analysis allowed the detection of both 3,4-DHPEA-EDA (oleacein) and p-HPEA-EDA (oleocanthal), but signals of 3,4-DHPEA-EA and p-HPEA-EA (oleuropein and ligstroside aglycones) were not detected. For these molecules, it is evident that there is not a homogeneous and linear dispersion of samples due to the particular clustering of T2 samples. In fact, the highest concentrations of 3, 4-DHPEA-EDA and p-HPEA-EDA were found in T2 oils (291 ± 5.6 and 205 ± 8.1 mg kg^−1^, respectively), derived from drupes which were characterized by a quite low ripening index (2.5 ± 0.14) and the lowest water content among all the studied fruits (61 ± 0.2) (Figures S6 and S7). Hence, the water content mainly differentiates the fruits and oils from T1 and T2, and the secoiridoids in the oils were negatively correlated with this factor. Similar to the secoiridoids in the fruits, the lowest values were reported for T3 and/or T4 oils (−86% for 3,4-DHPEA-EDA in T4 oils, −74% for p-HPEA-EDA in T3/T4 oils), confirming the negative (not statistically significant) correlation with the ripening index of the drupes (Figures S6 and S7). NMR analysis also allowed us to identify S-(E)-elenolide. Data indicated that there were no clear linear relationships between the ripening index and elenolide since there is not a homogeneous and linear dispersion of samples (Figure 4f). Similar to oleacein, the highest value was reported in T2 oils (112 ± 6.1 mg kg^−1^) and the lowest in T4 oils (−86%), in accordance with the negative correlation between the ripening index and water content of the drupes (Figure 4 and Figure S7).

### 2.5. Flavonoids and Lignan

**Figure 5:**
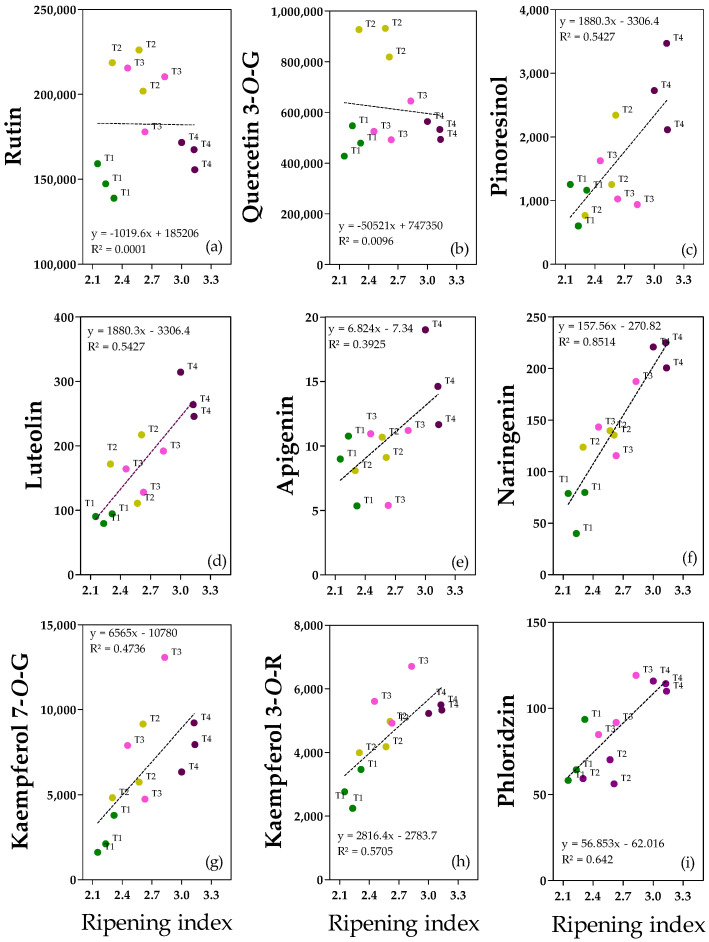
Regression analyses between ripening index and flavonoids and lignan (ng g^−1^ FW): (**a**) Rutin; (**b**) Quercetin-3-*O*-Glucoside (Quercetin-3-*O*-G); (**c**) Pinoresinol; (**d**) Luteolin; (**e**) Apigenin; (**f**) Naringenin; (**g**) Kaempferol-7-*O*-Glucoside (Kaempferol-7-*O*-G); (**h**) Kaempferol-3-*O*-Rutinoside (Kaempferol-3-*O*-R); (**i**) Phloridzin in fruits from olive (‘cv Arbequina’) trees subjected to T1 (•) full irrigation with tilled soil, T2 (•) reduced irrigation with tilled soil, T3 (•) full irrigation with non-tilled soil and, T4 (•) reduced irrigation with non-tilled soil.

The most relevant and significant flavonoids and lignans are shown in Figure 5, and clear linear relationships between the ripening index and pinoresinol, luteolin, apigenin; naringenin, kaempferol-7-*O*-Glucoside, kaempferol-3-*O*-Rutinoside and, phloridzin are reported (Figure 5c–i). The only exceptions were rutin and quercetin-3-*O*-Glucoside since there is not a homogeneous and linear dispersion of the data (Figure 5a,b).

Rutin (Figure 5a) and quercetin-3G (Figure 5b) were considered among the quercetin derivatives. Their concentrations ranged from 139 to 226 mg kg^−1^ for rutin and 427 to 931 mg kg^−1^ for quercetin-3G. Even if the correlation with pomological traits was not significant, rutin appeared to be more abundant in T2/T3 fruits than in T1/T4 fruits (−25%), suggesting that an intermediate ripening index, independent of the water content, promotes rutin abundance (Figure S8). Quercetin-3G showed a negative correlation with pericarp water content (as also shown in the regression analysis), and the highest concentration was reported in T2 fruits (which had the lowest water content) compared with T1, T3, and T4 fruits (+47%) (Figure S7). Among the flavones, luteolin (Figure 5d) and apigenin (Figure 5e) have been reported. Both were abundant in ripe fruits (0.3 ± 0.03 mg kg^−1^ for luteolin and 0.02 ± 0.003 mg kg^−1^ for apigenin), confirming the positive correlation with ripening degree. In the case of luteolin, the differences among treatments were significant, with ripe fruits being the richest in luteolin compared to the other treatments (+50%) (Figure S8). Furthermore, in the case of naringenin (Figure 5f), ripe fruits T4 reported the highest concentration (0.22 ± 0.01 mg kg^−1^) compared to T2/T3 (+33%) and to T1 (+67%) fruits. Regarding kaempferol derivates, T3 and T4 fruits had the highest kaempferol-3R concentration (5.7 ± 0.74 and 5.3 ± 0.11 mg kg^−1^ for T3 and T4 fruits, respectively), about +49% with respect to less ripe fruits (T1) (Figure S8). In kaempferol-7G, a positive correlation with the degree of ripening was observed (Figure 5g). Dihydrochalcone phloridzin was present in trace (0.06–0.12 mg kg^−1^), and it was more abundant in ripe fruits compared to T2 fruits (+45%), in accordance with the positive correlation with ripening degree (Figure 5i). There is no clear explanation for the difference between the T1 and T2 fruits. Concerning lignans, pinoresinol concentration ranged from 0.6 to 3.5 mg kg^−1^, and the highest amount was reported for ripe fruits compared to T1 and T3 ones (+61%) (Figure S8). It is worth noting that T2 fruits showed intermediate values between T1/T3 and T4 fruits.

### 2.6. Pigments

**Figure 6:**
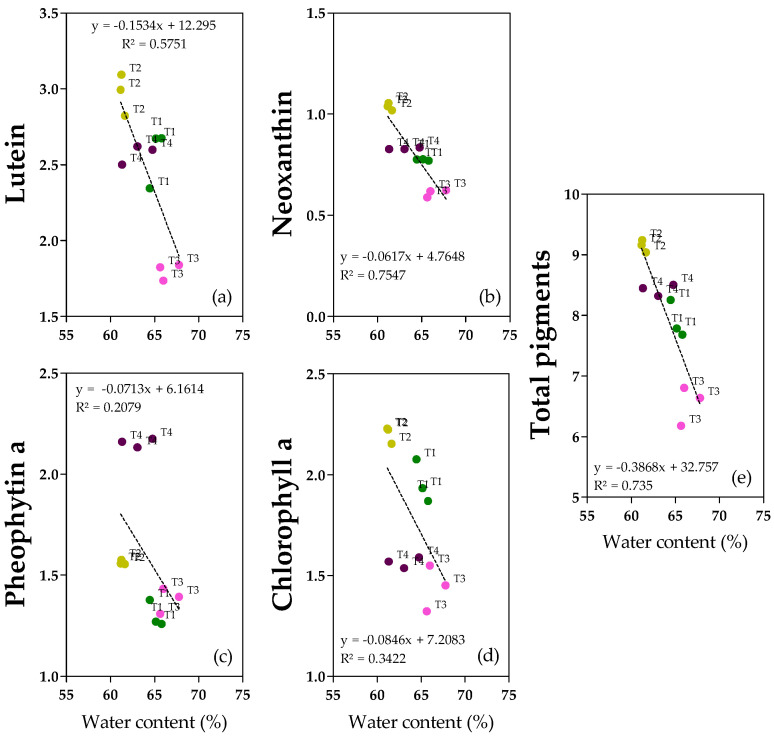
Regression analyses between water content (%) and pigments (mg kg^−1^ oil): (**a**) Lutein; (**b**) 9-cis Neoxanthin; (**c**) pheophytin *a*; (**d**) chlorophyll *a*; (**e**) total pigments in oils from olive (‘cv Arbequina’) trees subjected to T1 (•) full irrigation with tilled soil, T2 (•) reduced irrigation with tilled soil, T3 (•) full irrigation with non-tilled soil and, T4 (•) reduced irrigation with non-tilled soil.

Pigments in the oil samples were monitored. Lutein, pheophytin a and chlorophyll a (Figure 6a,c,d and Figure S9) were the most abundant, ranging from 1.7 to 3.1 mg kg^−1^ for lutein, and from 1.3 to 2.2 mg kg^−1^ from both pheophytin a and chlorophyll a. Neoxanthin (Figure 6b) was less represented (0.6–1.1 mg kg^−1^). Total pigments (Figure 6e and Figure S9) ranged from 6.2 to 9.2 mg kg^−1^. In accordance with the negative correlation with fruit water content and with the regression analyses, lutein, neoxanthin, and total pigments showed the highest concentrations in T2 oils and the lowest in T3 oils (+39% for both lutein and neoxanthin, +29% for total pigments) (Figure S9). Regarding chlorophyll a, T3 and T4 oils showed similar low concentrations, whereas both T1 and T2 oils were richer in chlorophyll a (+25% and +32%, respectively), confirming both the correlation with the degree of ripening (y = −0.5598x + 3.2551, R^2^ = 0.3501, *p* = 0.0009) (Figures S9 and S10) and with water content (Figure 6). Pheophytin a showed a different behavior compared to chlorophyll a. In fact, there were no clear linear relationships with water content since there is not a homogeneous and linear dispersion of samples also observed for the regression with the degree of ripening (y = 0.8462x − 0.6108, R^2^ = 0.6848, *p* = 0.0427) (Figure S10); T4 oil showed the highest concentration (+37% with respect to T1/T3 and +16% with respect to T2) (Figure S9). Interestingly, chlorophyll a and pheophytin a showed the opposite behavior with respect to the ripening degree (Figure S10).

## 3. Discussion

A different agronomic background allowed us to mimic real consistent changes in fruit traits, as well as in the composition of drupes and, consequently, of the oils. Olive fruit traits, especially the ripening degree, are usually related to the abundance of minor components in olive fruits and oils. The differences observed in pomological traits were minor. For instance, the ripening index varied between 2.2 and 3.1, which corresponds to different steps of the turning phase (reddish spots on ≤50% of the whole fruit epicarp = 2; reddish spots on ≥50% of the whole fruit epicarp). However, these changes were consistent with those reported by García-González et al. [21], who compared various soil management practices (tillage, spontaneous vegetation, *Brachypodium*, and bitter vetch). In their study, the maturity index of olive fruits varied between 1.9 and 2.9 among treatments. García et al. [24] reported changes in the ripening index (ranging between 2 and 4 in the early harvest, although the difference among treatments was not significant) and in the color index in fruits of the olive tree cultivar, Arbequina, subjected to different irrigation regimes and strategies. Similar to our results, the same authors observed significant changes in pericarp water content (ranging between 51% and 63% during early harvesting).

Modifications in fruit characteristics were well correlated with minor components, allowing us to study the relationship between ripening degree, water content, and specific phenolic compounds and pigments in both fruits and oils. As a general consideration, we detected low amounts of phenols (especially secoiridoids) and pigments, but it is known that the environmental conditions of a specific cultivation area could have been relevant [27]. We found a negative regression and correlation between the degree of ripening and phenolic alcohols, secoiridoids (both in fruit and oil), and most phenolic acids and their derivatives (i.e., verbascoside), while a positive correlation with flavonoids (and lignan) has been reported. The general decrease observed in important phenolic compounds (oleuropein, 3,4-DHPEA, and verbascoside) in Arbequina throughout ripening has been described by various authors [15,18,28].

The decline in simple phenols (most of the phenolic acids and their derivatives) could be related to phenylalanine ammonia lyase (PAL) activity, an enzyme that catalyzes the first committed step in phenylpropanoid biosynthesis. PAL activity decreases over ripening [29] and is influenced by agricultural practices (i.e., irrigation management). The decline in PAL activity was correlated with a reduction in phenolic compounds [22,30]. In contrast, Alagna et al. [31] observed an increase in PAL gene expression 165 days after flowering, coincidently with the synthesis and accumulation of anthocyanins in the drupe. Consistent with this observation, the set of flavonoids we studied (naringenin, luteolin, apigenin, rutin, quercetin-3G, kaempferol-7G, kaempferol-3R, and phloridzin) was positively correlated with the degree of ripening. This finding is in accordance with the results of De Torres et al. [32], who reported the highest flavonoid concentration in ripe fruits (RI = 4). Some flavonoids identified in our study, such as quercetin-3G and naringenin, are not usually reported in olive products. Naringenin was reported in Argentinian (cv Aruco and Empeltre) monovarietal olive oils [33] and in oleaster oils (produced from wild olive tree fruits) [34]. Traces of naringenin were also found in olive fruit and pomace [35], demonstrating that its content can be affected by the pressing procedure; however, to the best of our knowledge, its correlation with ripening has not yet been described. Compared to our results, a significant increase in luteolin and rutin was also observed by Artajo et al. [36] in olive paste (cultivar Arbequina) as the ripening index increased (from 2 to 6), while the apigenin concentration was quite constant. Apigenin and luteolin are derived from naringenin because of two enzymes (flavone synthase and flavone 3′hydroxylase), whereas rutin is derived from quercetin. Quercetin and kaempferol are synthesized from dihydrokaempferol, which is a crucial intermediate derived from naringenin. Apart from rutin, quercetin and kaempferol derivatives are not commonly studied in olive products, but some authors have observed that they can be involved in the stress response of olive leaves [37]. Considering both the increase in flavonoids and the decrease in phenolic acids, it seems that as ripening advances, more complex molecules derived from the phenylpropanoid pathway are favoured. In fact, the same anthocyanins are produced from dihydroquercetin, which originates from dihydrokaempferol. Concerning pinoresinol and phloridzin, both compounds derive from the first products of the phenylpropanoid pathway (p-coumaric acid and p-coumaroyl CoA, respectively) and were positively correlated with ripening degree. Consistent with our observations, Alagna et al. [31] detected a modest increase in pinoresinol during ripening. Thus, while the precursor p-coumaric acid is negatively correlated with the ripening degree, pinoresinol, a final product (similar to flavonoids), accumulates in more ripe olive fruits. Phloridzin was putatively identified in olive oil [38]; however, to the best of our knowledge, it has not been reported in olive fruits. In fact, it is worth noting that UHPLC-MS/MS analysis of fruit phenols allowed us to identify various trace compounds (not all correlated with fruit traits). This is the case of the flavonoid myricetin. This compound, which is also derived from dihydrokaempferol, has already been studied in olive fruits by Niu et al. [39] and Ahmad et al. [40]. In both cases, the intensity of the signal was too low to be detected. However, both the authors worked on completely ripe fruits (RI = 4). Conversely, to the best of our knowledge, the kaempferol derivative tiliroside has been studied and quantified only in olive leaves (with concentrations ranging from 6 to 15 mg kg^−1^ DW) [41].

Unlike simple phenols and flavonoids, in olive fruit, secoiridoids (formed by a terpenic and a phenolic moiety) are putatively produced from two different metabolic pathways: the terpenic moiety is generated from geraniol, which is derived from the plastidic MEP pathway, whereas the phenolic moiety is derived from L-arogenate, which is converted into tyrosine, the substrate for both p-HPEA and 3,4-DHPEA production [31]. In agreement with our results, the same authors observed that most genes involved in both terpenic and phenolic moiety formation were downregulated during ripening. Concerning olive oil typical secoiridoids, in the same study, the role of 3,4-DHPEA-EDA as a potential precursor of oleuropein biosynthesis was also discussed, suggesting that part of the 3,4-DHPEA-EDA found in the oil could derive directly from the fruit. At the same time, it was demonstrated that the peroxidase (POX) activity (especially detected in the seed) has a role in the conversion of oleuropein in 3,4-DHPEA-EDA during olive oil extraction [5]. POX activity was quite constant at harvest time (28 weeks after flowering), suggesting that the initial abundance of the substrate (oleuropein) in our fruit samples could have played a role in 3,4-DHPEA-EDA abundance in the produced oil. Interestingly, we found a stronger negative correlation between oil secoiridoids and pericarp water content than between oil secoiridoids and ripening degree. A significant role of pulp water content in relation to the amount of phenolic compounds was also observed by Benito et al. [42]. The authors hypothesized that, as phenolic compounds are more soluble in water than in oil, the reduced water content in the pericarp could improve the extractability of phenolic compounds from the olive fruit and their allocation in the oil phase. This hypothesis could explain the differences observed between T1 and T2 and between T3 and T4 oils in terms of 3,4-DHPEA-EDA and p-HPEA-EDA concentrations. NMR analysis allowed us to identify and quantify S-(E)-Elenolide [26]. In accordance with its putative origin (oleuropein and ligstroside), this compound showed similar behavior to 3,4-DHPEA-EDA and p-HPEA-EDA, being negatively correlated with ripening degree and water content in the pericarp.

Similar to secoiridoids in the oil, most of the pigments showed a negative correlation with pericarp water content and, to a lesser extent, with the ripening degree. According to the hypothesis of Benito et al. [42], olive pastes with a higher water content are more fluid and suffer less tissue crushing owing to the higher speed during hammer–crush crossing. For this reason, consistent with our results, less pigment was extracted from the paste and transferred to the oil. This effect seems to be more evident in xanthophylls (lutein and 9-cis neoxanthin) than in chlorophylls. It should be noted that the total amount of pigments in all the oil samples investigated here was slightly lower than what is normally found [43]. With respect to ripening, both chlorophyll *a* and pheophytin *a* were significantly affected by ripening degree, whereas for carotenoids, the correlation was not significant (and the correlation coefficient was close to zero). This observation is consistent with previous studies on the cultivar Arbequina, and it was demonstrated that the loss of chlorophyll during ripening is significantly higher than that during carotenoid degradation [19]. Our data indicated that chlorophyll *a* and pheophytin *a* showed the opposite behavior with respect to the ripening degree, in agreement with the hypothesis that chlorophyll *a* is degraded to pheophytin *a* during ripening.

## 4. Materials and Methods

### 4.1. Experimental Material

The experiment was conducted in 2019 in a 20 years-old super-intensive olive orchard (*Olea europaea* L., cv Arbequina) located in Marina di Grosseto (42.735394 N, 10.986208 E), Grosseto, Italy. The plants were trained as hedgerows with 1562 trees per hectare at a spacing of 4 *×* 1.6 m.

The orchard had a drip irrigation system, and it was divided into five different plots, each composed of 17–20 plant rows covering a total of 1.22–1.44 hectares. Fruit and oil samples from four of these plots were selected for this study.

i. *T1: Full irrigation with tilled soil.*
ii. *T2: Reduced irrigation with tilled soil.*
iii. *T3: full irrigation with non-tilled soil.*
iv. *T4: Reduced irrigation with non-tilled soil.*

Fully irrigated plants received 100% of the water needs (obtained from previous studies on the water needs of olive trees in the same environment [44]), while trees subjected to reduced irrigation received 35% of the water needs. Water was supplied once every two weeks, from the 15th of June to the 16th of September. In the tilled plots, the soil was tilled using a disc harrow in April, June, and October.

To avoid border effects, only the central rows and inner plants were used to collect olive samples. Olive fruits (approximately 40 kg) were harvested by hand at harvest time, immediately before milling at the end of October. After harvesting, the olives were washed and crushed using a two-phase Oliomio^®^ continuous mill (Toscana Enologica Mori, Tavarnelle, Italy). The system reproduces, at a small scale, the industrial method of oil extraction so that the resulting oils are similar to those produced at a commercial scale, but the extraction, especially referring to the low addition of water during the transport to the centrifuge, was carried out to obtain an optimal recovery of MPCs. All operational conditions were kept constant for all samples: the temperature was fixed at 25–27 °C, the time of malaxation was 30 min, the flux of water in the separator was 2 L/h, and the speed of the centrifuge was 3800 rpm. The extractions were performed by a single operator. The oils at the exit of the horizontal centrifuge were immediately filtered using a cotton laboratory filter and divided into 100 mL sample bottles.

### 4.2. Fruit Traits

Some of the harvested olive fruits were used to determine fruit traits and phenolic compounds in the pericarp. The ripening index was determined according to the Jaen index [45] on 50 fruits. The fresh weight of a single fruit was determined by weighing 10 fruits and calculating the average weight of a single fruit. The pericarp was separated from the endocarp, and the pulp/pit ratio was determined. The water content in the pericarp was obtained using the following equation: (FW-DW)/FW × 100, where FW corresponds to the fresh weight, and DW, the dry weight, was obtained after incubation at 60 °C until a constant weight was reached. The oil content in the pericarp was determined through the use of Soxhlet extraction with petroleum ether (VWR Chemicals, Fontenay-sous-Bois, France) for 8 h. All analyses were performed in triplicate.

### 4.3. Phenolic Compounds Determination in Olive Fruit

The phenolic compounds were extracted according to the method described by Machado et al. [30]. Approximately 5 g of pulverised olive pericarp (*n* = 3) was extracted in 50 mL of a 50:50 (*v*/*v*) methanol/water solution and shaken in the dark for 1.5 h at room temperature (RT). The extracts were then filtered and washed twice with hexane (3:1). Finally, an aliquot of the methanol: water phase was filtered through a Whatman (Puradisc, 0.45 µm) cartridge and diluted before UHPLC-MS/MS analysis.

A Sciex 5500 QTrap^+^ mass spectrometer (AB Sciex LLC, Framingham, MA, USA) equipped with a Turbo V ion spray source coupled to an ExionLC AC System custom-made by Shimadzu (Shimadzu Corporation, Kyoto, Japan) was used to study 41 known phenols. More in detail, 9 phenolic acids (2-coumaric acid, 3-coumaric acid, 4-coumaric acid, vanillic acid, gallic acid, caffeic acid, cinnamic acid, protocatechuic acid and *trans*-ferulic acid), 5 caffeic acid derivates (cynarine, rosmarinic acid, chlorogenic acid, chicoric acid and verbascoside), 2 phenolic alcohols (tyrosol and hydroxytyrosol), 2 secoiridoids (oleuropein and ligstroside), 10 flavonols and their derivates (quercetin, quercetin-3-*O*-Glucoside, quercetin-3,4′-*O*-DiGlucoside, rutin, quercetagenin-7-*O*-Glucoside, myricetin, kaempferol-3-*O*-Rutinoside, kaempferol-7-*O*-Glucoside, kaempferol-3-*O*-Glucoside and tiliroside), 1 flavanone (naringenin), 2 flavones (apigenin and luteolin), 1 lignan (pinoresinol), 2 flavanols (catechin and epicatechin), 3 procyanidin (procyanidin B1, procyanidin B2 and procyanidin B3), 2 stilbenoids (piceid and resveratrol) and 2 dihydrochalcones (phloretin and phloridzin) were analyzed. The common source parameters, SRM transitions, and corresponding compound parameters are reported in the Supplementary Material (Table S4).

A Phenomenex Kinetex ^®^ Biphenyl 100 × 2.1 mm, 2.6 µm particle size column (Phenomenex, Torrance, CA, USA) was used for chromatographic separation. An elution gradient was performed using acetonitrile containing 0.1% formic acid and Milli-Q water with 0.1% formic acid. 

### 4.4. Secoiridoids Quantification in Olive Oil

Secoiridoids were determined using ^1^H NMR spectroscopy according to the protocol proposed by Karkoula et al. [46] and slightly modified by Vicario et al. [47]. Briefly, approximately 4 g of olive oil was added to 20 mL of hexane and shaken thrice. Then, 25 mL of acetonitrile was added, shaken three times, and left until the two phases were separated. Finally, the acetonitrile fraction was collected, and 500 µL of syringaldehyde (analytical standard, IUPAC name:4-hydroxy-3,5-dimethoxybenzaldehyde, Sigma-Aldrich, St. Louis, MO, USA) was added as an internal standard, and the mixture was evaporated under nitrogen flow. The residue was dissolved in 500 µL of deuterated chloroform (CDCl_3_) and transferred to an NMR tube. ^1^H NMR spectra of the extracts were recorded by using a 30° pulse on the ^1^H channel of 3.9 μs at 29.92 W (90° pulse is 11.8 μs), with an acquisition time of 2 s (32 K points zero filled to 64 K before Fourier Transform) and a relaxation delay of 1.5 s. The number of scans was 400 for approximately 16 min. The line broadening was set at 0.5 Hz, the phase was adjusted automatically, and the baseline was corrected manually in the region of interest. The spectra were normalised. The peaks were selected manually based on the literature data [46]. The peak area was determined via the deconvolution of the signals. Quantification of the identified compounds was performed according to Karkoula et al. [46]. NMR analysis allowed us to identify and quantify S-(E)-Elenolide, as previously described in the literature [26].

### 4.5. Pigments Determination in Olive Oil

Analysis of near UV-Vis absorbance spectra was performed to determine the main pigments (β-carotene, lutein, 9-cis-neoxantin, pheophytin *a*, and chlorophyll *a*) using the method described by Domenici et al. [48] and validated by Lazzerini et al. [43]. No sample treatment was required, as oil samples (n = 3) were analyzed directly in the bulk. In particular, the samples were placed in a transparent quartz cuvette with an optical path of 0.5 cm. The spectra were recorded for each sample in triplicate between 390 and 720 nm with a resolution of 1 nm using a Jasco UV-Vis V-550 double-beam spectrophotometer. The spectral baseline was corrected to fix the absorbance at 720 nm at zero before spectral deconvolution for pigment quantification.

### 4.6. Statistical Analysis

All data are expressed as mean ± standard deviation (SD). Differences between means in the four treatments were assessed using one-way ANOVA. Tukey’s post hoc test was applied at *p* = 0.05. Principal components analysis (PCA) was performed to detect the relationships between variables. Graphs were generated using Prism5 (GraphPaD). Correlations among parameters were analyzed using Spearman’s correlations. Statistical analyses were performed in R Studio (2022.12.0 + 353).

## 5. Conclusions

In conclusion, our study points out that at harvest time, the ripening degree and pericarp water content correlate with the abundance of phenols and pigments in both olive fruit and oil samples derived from different agronomic backgrounds. The positive or negative correlation between the degree of ripening and fruit phenols likely depends on the specific metabolic pathway that leads to the formation of each individual phenol derivative. Conversely, secoiridoids and pigments in the oil are affected more by pericarp water content, as this factor affects the extractability of most of these minor components.

## Data Availability

The data supporting the findings of this study are available from the corresponding author upon reasonable request.

## Supplementary Materials

The following supporting information can be downloaded at: https://www.mdpi.com/article/10.3390/molecules28196943/s1, Supplementary Materials. Figure S1. PCA Biplot of Dim1 vs Dim2 showing loadings of the scores and the variables water content, ripening index, phenolic acids and derivatives. Figure S2. PCA Biplot for PC1 (Dim1) and PC2 (Dim2) showing loadings of the scores and the variables water content, ripening index, phenolic alcohols secoiridoids and derivatives. Figure S3. PCA Biplot for PC1 (Dim1) and PC2 (Dim2) showing loadings of the scores and the variables water content, ripening index, flavonoids and lignan. Figure S4. PCA Biplot for PC1 (Dim1) and PC2 (Dim2) showing loadings of the scores and the variables of water content, ripening index, and pigments. Table S1. Correlation table among fruit pomological traits and fruit phenolic compounds and secoiridoids (and derivates) and pigments in the oil. Table S2. *p*-values associated to the correlation coefficients shown in Table SM1. Table S3. Mean values ± standard deviations of fruit phenolic compounds. Figure S5. Mean values ± standard deviations of phenolic acids and derivates. Figure S6. Mean values ± standard deviations of phenolic alcohols, secoiridoids and derivates. Figure S7. Regression analysis between water content (%) and (a) Quercetin-3-*O*-Glucoside (Quercetin-3-*O*-G) (ng g^−1^ FW) in fruits. Figure S8. Mean values ± standard deviations of flavonoids and lignan. Figure S9. Mean values ± standard deviations of pigments. Figure S10. Regression analyses between ripening index pigments in oils. Table S4. SRM transitions and corresponding phenols’ parameters.
