# Supplementary material for: Olive Fruit Ripening Degree and Water Content Relationships with Phenolic Acids and Alcohols, Secoiridoids, Flavonoids and Pigments in Fruit and Oil
Source: Molecules. 2023 Oct 5;28(19):6943. doi: 10.3390/molecules28196943 (PMC10574279; doi:10.3390/molecules28196943)
Supplement: Supplementary file 1 [file molecules-28-06943-s001.zip › molecules-2622738-supplementary.pdf]

Supplementary Materials

Results

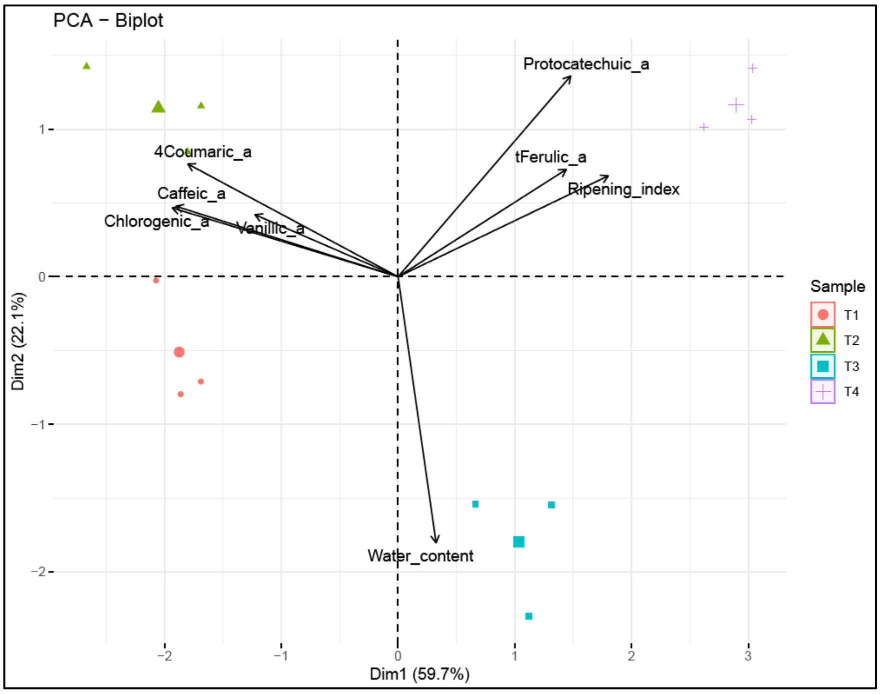

Figure S1. PCA Biplot of Dim1 vs Dim2 showing loadings of the scores and the variables water content, ripening index, 4-coumaric acid (4Coumaric_a), caffeic acid (Caffeic_a), *trans*-ferulic acid (tFerulic_a), vanillic acid (Vanillic_a), protocatechiuc acid (Protocatechuic_a), chlorogenic acid (Chlorogenic_a), in fruits from olive ('cv Arbequina') trees subjected to T1 (•) full irrigation with tilled soil, T2 (•) reduced irrigation with tilled soil, T3 (•) full irrigation with non-tilled soil and, T4 (•) reduced irrigation with non-tilled soil.

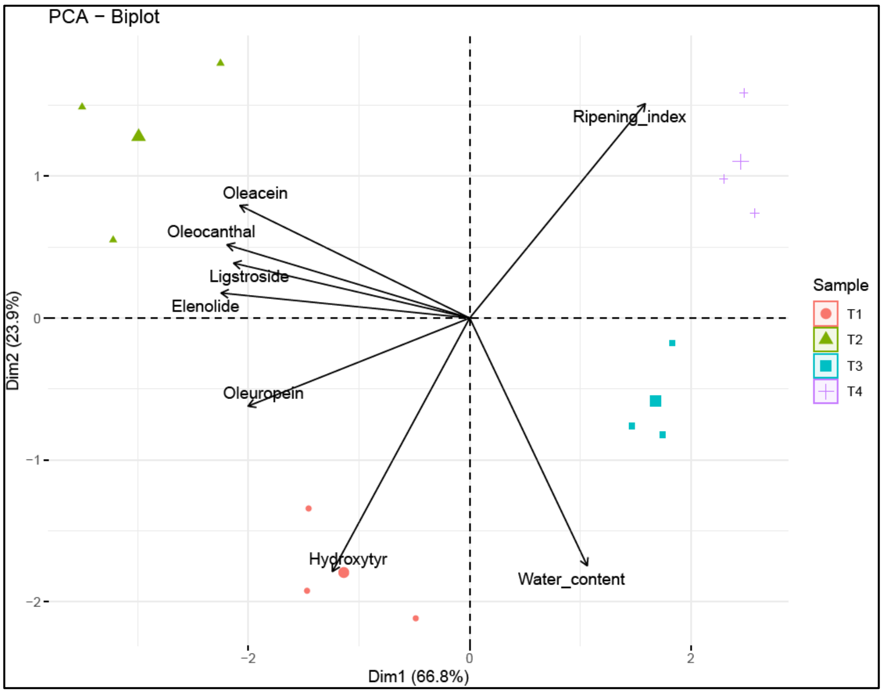

Figure S2. PCA Biplot for PC1 (Dim1) and PC2 (Dim2) showing loadings of the scores and the variables water content, ripening index, 3,4-DHPEA (Hydroxytyr), oleuropein, ligstroside, (monitored in fruits) 3,4-DHPEA-EDA (Oleacein), p-HPEA-EDA (Oleocanthal), and S-(E)-elenolide (monitored in oils) from olive ('cv Arbequina') trees subjected to T1 (•) full irrigation with tilled soil, T2 (•) reduced irrigation with tilled soil, T3 (•) full irrigation with non-tilled soil and, T4 (•) reduced irrigation with non-tilled soil.

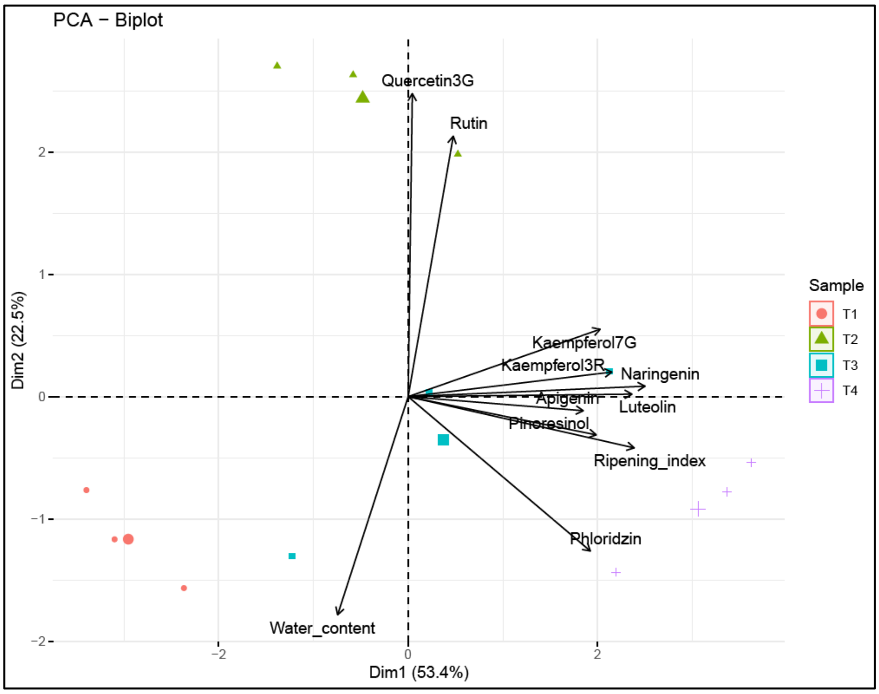

Figure S3. PCA Biplot for PC1 (Dim1) and PC2 (Dim2) showing loadings of the scores and the variables water content, ripening index, Rutin, Quercetin-3-O-Glucoside (Quercetin3G), pinoresinol, luteolin, apigenin, naringenin, kaempferol-7-O-Glucoside (Kaempferol7G), kaempferol-3-O-Rutinoside (Kaempferol3R), phloridzin in fruits from olive ('cv Arbequina') trees subjected to T1 (•) full irrigation with tilled soil, T2 (•) reduced irrigation with tilled soil, T3 (•) full irrigation with non-tilled soil and, T4 (•) reduced irrigation with non-tilled soil.

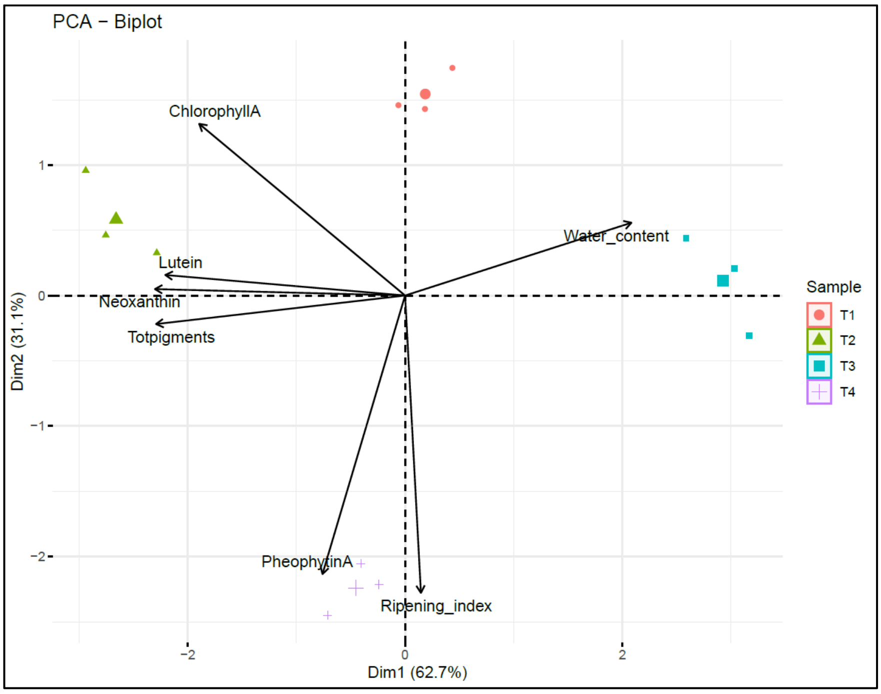

Figure S4. PCA Biplot for PC1 (Dim1) and PC2 (Dim2) showing loadings of the scores and the variables of water content, ripening index, lutein, 9-cis Neoxanthin (Neoxanthin), Pheophytin *a* (PheophytinA), Chlorophyll *a* (ChlorophyllA), Total pigments (Totpigments) in oils from olive ('cv Arbequina') trees subjected to T1 (•) full irrigation with tilled soil, T2 (•) reduced irrigation with tilled soil, T3 (•) full irrigation with non-tilled soil and, T4 (•) reduced irrigation with non-tilled soil.

Table S1. Correlation table among fruit pomological traits and fruit phenolic compounds and secoiridoids (and derivates) and pigments in the oil.

	Ripening_index	FW	Pulppit_ratio	Water_content	Oil_content
**Ripening_index**		0.427	-0.028	-0.098	0.070
**FW**	0.427		0.343	-0.350	0.175
**Pulppit_ratio**	-0.028	0.343		0.322	-0.224
**Water_content**	-0.098	-0.350	0.322		-0.580
**Oil_content**	0.070	0.175	-0.224	-0.580	
**Protocatechuic_a**	0.734	0.476	-0.196	-0.510	0.245
**Vanillic_a**	-0.483	-0.524	-0.643	-0.196	0.448
**Caffeic_a**	-0.650	-0.210	0.028	-0.329	0.524
**4Coumaric_a**	-0.685	-0.357	-0.266	-0.329	0.462
**tFerulic_a**	0.308	0.126	-0.434	0.098	0.021
**Chlorogenic_a**	-0.671	-0.238	-0.056	-0.350	0.399
**Chicoric_a**	0.385	0.077	-0.231	-0.608	0.545
**Verbascoside**	-0.860	-0.336	-0.007	-0.077	0.308
**p-HPEA**	0.291	0.067	-0.291	-0.343	-0.097
**3,4-DHPEA**	-0.909	-0.371	0.007	0.126	0.105
**Ligstroside**	-0.552	-0.238	0.049	-0.371	0.308
**Oleuropein**	-0.769	-0.378	-0.077	-0.175	0.252
**Naringenin**	0.860	0.748	0.070	-0.266	0.084
**Apigenin**	0.650	0.552	-0.119	-0.203	-0.126
**Luteolin**	0.839	0.510	-0.091	-0.294	0.259
**Catechin**	-0.245	-0.503	0.385	0.287	-0.077
**Epicatechin**	0.035	-0.259	0.238	-0.049	0.000
**Quercetin**	0.287	0.070	0.042	0.119	-0.063
**Myricetin**	0.077	0.427	0.098	0.028	0.021
**Quercetin3G**	0.105	0.448	0.119	-0.748	0.420
**Quercetagetin7G**	0.364	-0.147	-0.455	-0.189	-0.042
**Quercetin34DG**	0.112	0.650	0.748	-0.259	0.175
**Rutin**	0.028	0.490	0.664	-0.280	0.014
**Kaempferol7G**	0.769	0.699	0.245	-0.210	0.308
**Kaempferol3G**	0.490	0.790	0.378	-0.210	0.231
**Kaempferol3R**	0.762	0.706	0.406	0.126	-0.028
**Tiliroside**	0.420	0.224	0.049	0.077	0.280
**Pinoresinol**	0.580	0.273	-0.329	-0.168	-0.056
**Phloridzin**	0.720	0.497	0.014	0.154	-0.098
	**Ripening_index**	**FW**	**Pulppit_ratio**	**Water_content**	**Oil_content**
**Ripening_index**		0.427	-0.028	-0.098	0.070
**FW**	0.427		0.343	-0.350	0.175
**Pulppit_ratio**	-0.028	0.343		0.322	-0.224
**Water_content**	-0.098	-0.350	0.322		-0.580
**Oil_content**	0.070	0.175	-0.224	-0.580	
**3,4-DHPEA-EDA**	-0.671	-0.287	0.021	-0.322	0.357
**p-HPEA-EDA**	-0.657	-0.315	-0.014	-0.301	0.329
**Elenolide**	-0.706	-0.343	-0.014	-0.308	0.378
**Lutein**	-0.294	-0.203	-0.336	-0.678	0.545
**bCarotene**	0.070	0.154	-0.021	-0.259	-0.077
**Neoxanthin**	0.077	0.063	-0.399	-0.860	0.608
**PheophytinA**	0.671	0.476	-0.161	-0.580	0.245
**ChlorophyllA**	-0.510	-0.154	-0.287	-0.671	0.503
**Totpigments**	0.049	0.147	-0.350	-0.895	0.601

Table S2. P-values associated to the correlation coefficients shown in Table S1.

	p.Ripening_index	p.FW	p.Pulppit_ratio	p.Water_content	p.Oil_content
**Ripening_index**		0.09934	0.74767	0.56027	0.76491
**FW**	0.09934		0.37967	0.19846	0.65824
**Pulppit_ratio**	0.74767	0.37967		0.13938	0.74516
**Water_content**	0.56027	0.19846	0.13938		0.07653
**Oil_content**	0.76491	0.65824	0.74516	0.07653	
**Protocatechuic_a**	0.00041	0.21412	0.09368	0.15136	0.60897
**Vanillic_a**	0.04316	0.04086	0.10787	0.83151	0.45494
**Caffeic_a**	0.01344	0.33130	0.82278	0.22875	0.15419
**4Coumaric_a**	0.01654	0.29448	0.45254	0.18350	0.08246
**tFerulic_a**	0.02552	0.76741	0.11870	0.93755	0.91909
**Chlorogenic_a**	0.00580	0.26507	0.52837	0.29318	0.22343
**Chicoric_a**	0.22905	0.92803	0.46530	0.13670	0.05966
**Verbascoside**	0.00523	0.34407	0.63815	0.67545	0.21363
**p-HPEA**	0.11341	0.75245	0.15633	0.43579	0.76097
**3,4-DHPEA**	0.00031	0.06208	0.81467	0.46421	0.94179
**Ligstroside**	0.08801	0.82683	0.79600	0.10804	0.35093
**Oleuropein**	0.00934	0.53780	0.62222	0.41396	0.42492
**Naringenin**	0.00002	0.02109	0.98831	0.34459	0.67395
**Apigenin**	0.02926	0.11580	0.26289	0.23339	0.55771
**Luteolin**	0.00049	0.14511	0.42445	0.24468	0.49265
**Catechin**	0.34383	0.03869	0.30387	0.16224	0.41255
**Epicatechin**	0.81515	0.57714	0.44636	0.93925	0.69540
**Quercetin**	0.98832	0.93745	0.65829	0.91981	0.87746
**Myricetin**	0.62141	0.58168	0.86263	0.84988	0.82949
**Quercetin3G**	0.76162	0.30837	0.81904	0.00753	0.09822
**Quercetagetin7G**	0.11936	0.99842	0.10322	0.40967	0.80592
**Quercetin34DG**	0.87302	0.04863	0.24821	0.13708	0.45908
**Rutin**	0.97169	0.07254	0.06329	0.28685	0.67987
**Kaempferol7G**	0.01335	0.01992	0.16802	0.63474	0.29130
**Kaempferol3G**	0.62622	0.02105	0.55428	0.48985	0.96970
**Kaempferol3R**	0.00450	0.02476	0.14641	0.95896	0.70120
**Tiliroside**	0.13881	0.50036	0.21513	0.77267	0.25672
**Pinoresinol**	0.00628	0.27294	0.12468	0.18321	0.86143
**Phloridzin**	0.00173	0.11175	0.77133	0.51558	0.85380
	**p.Ripening_index**	**p.FW**	**p.Pulppit_ratio**	**p.Water_content**	**p.Oil_content**
**Ripening_index**		0.09934	0.74767	0.56027	0.76491
**FW**	0.09934		0.37967	0.19846	0.65824
**Pulppit_ratio**	0.74767	0.37967		0.13938	0.74516
**Water_content**	0.56027	0.19846	0.13938		0.07653
**Oil_content**	0.76491	0.65824	0.74516	0.07653	
**3,4-DHPEA-EDA**	0.16126	0.84028	0.81981	0.03434	0.12239
**p-HPEA-EDA**	0.07955	0.60135	0.62316	0.04858	0.15581
**Elenolide**	0.02293	0.37871	0.59347	0.10495	0.19279
**Lutein**	0.69255	0.63969	0.02141	0.00426	0.08367
**bCarotene**	0.78599	0.82420	0.66391	0.51567	0.72385
**Neoxanthin**	0.84561	0.97908	0.07671	0.00025	0.06776
**PheophytinA**	0.00089	0.21166	0.08985	0.13633	0.68977
**ChlorophyllA**	0.04272	0.42207	0.20896	0.04571	0.22701
**Totpigments**	0.96270	0.99597	0.01097	0.00036	0.12780

Table S3. Mean values ±standard deviations of fruit phenolic compounds (chicoric acid, p-HPEA, catechin, epicatechin, quercetin, myricetin, quercetagetin7G, quercetin3,4DG, kaempferol3G, tiliroside reported as ng g^{-1} FW) and pigments (β-Carotene, μg g^{-1} oil) in fruits from olive ('cv Arbequina') trees subjected to T1, T2, T3 and T4. Data were analyzed by one-way ANOVA. P-values are reported in the Table. Abbreviations: G=glucoside, DG=diglucoside.

Compound	T1	T2	T3	T4	P-value
Chicoric acid	48±11.9	146±85.0	66±47.2	126±47.7	0.212
p-HPEA	1688±1472.3	919±1591.4	nd	3879±1566.7	0.055
Catechin	131±60.7	120±37.1	143±38.7	105±3.1	0.736
Epicatechin	110±44.7	125±28.2	127±46.5	106±17.1	0.897
Quercetin	65±36.8	501±582.4	597±385.8	234±101.6	0.359
Myricetin	224±91.4	226±215.8	286±63.6	348±156.5	0.741
Quercetagetin7G	33±11.5	31±9.9	28±9.1	53±11.6	0.118
Quercetin3,4DG	43±7.8	89±30.3	66±8.1	52±9.4	0.053
Kaempferol3G	52±33.5	1083±1606.3	1995±2490.6	2004±2598.4	0.712
Tiliroside	77±30.2	99±61.8	138±63.7	118±34.9	0.605
b-Carotene	1.3±0.32	1.4±0.10	1.3±0.14	1.3±0.08	0.962

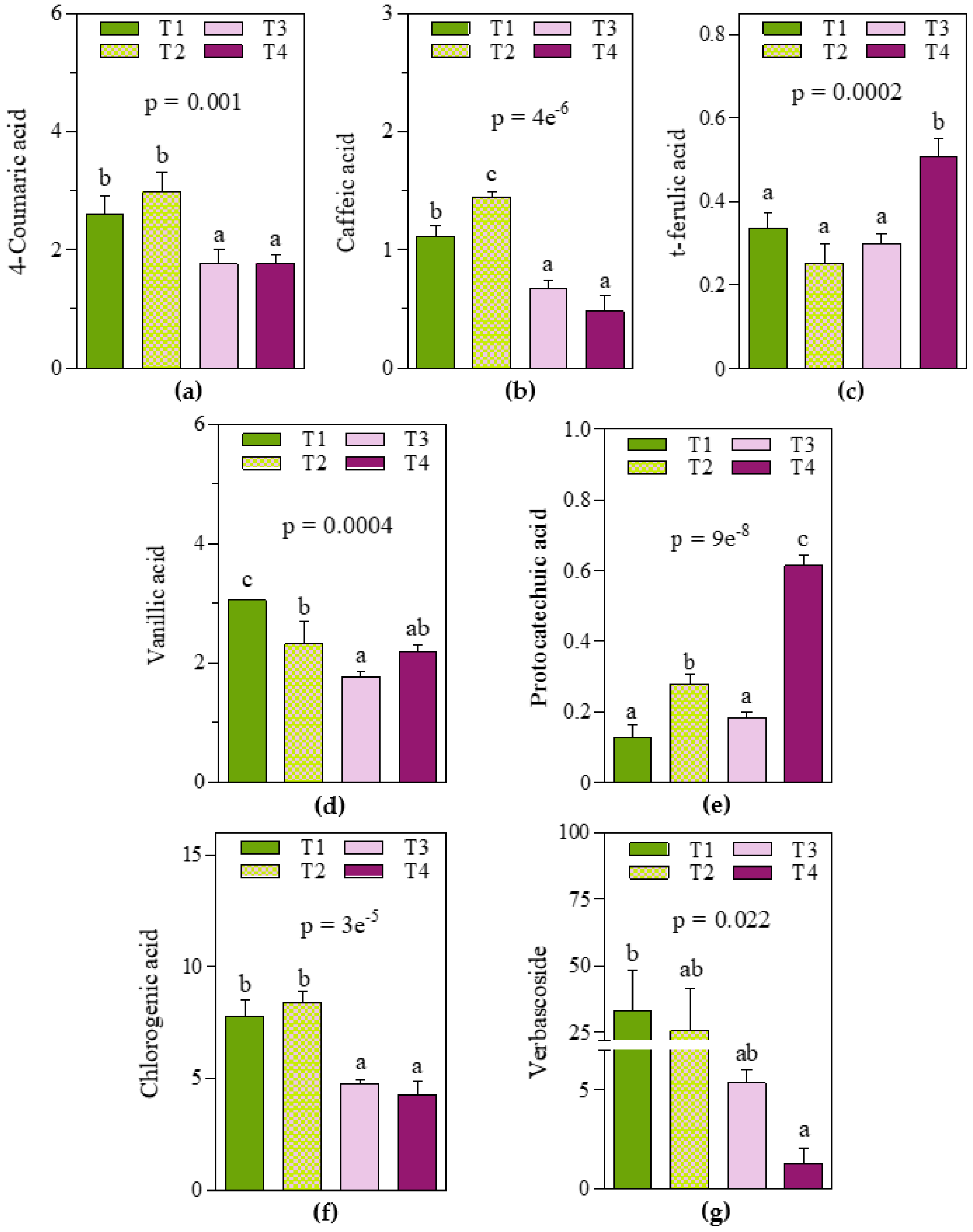

Figure S5. Mean values ± standard deviations of phenolic acids and derivates (mg kg^{-1} FW): **(a)** 4-coumaric acid; **(b)** Caffeic acid; **(c)** trans-ferulic acid (t-ferulic acid); **(d)** Vanillic acid; **(e)** Protocatechiuc acid; **(f)** Chlorogenic acid; **(g)** Verbascoside in fruits from olive ('cv Arbequina') trees subjected to T1, T2, T3 and T4. Data were analyzed by one-way ANOVA Tukey's post-hoc test at 0.05 probability level was applied and different letters indicated significant difference. P-values are reported in the graphs.

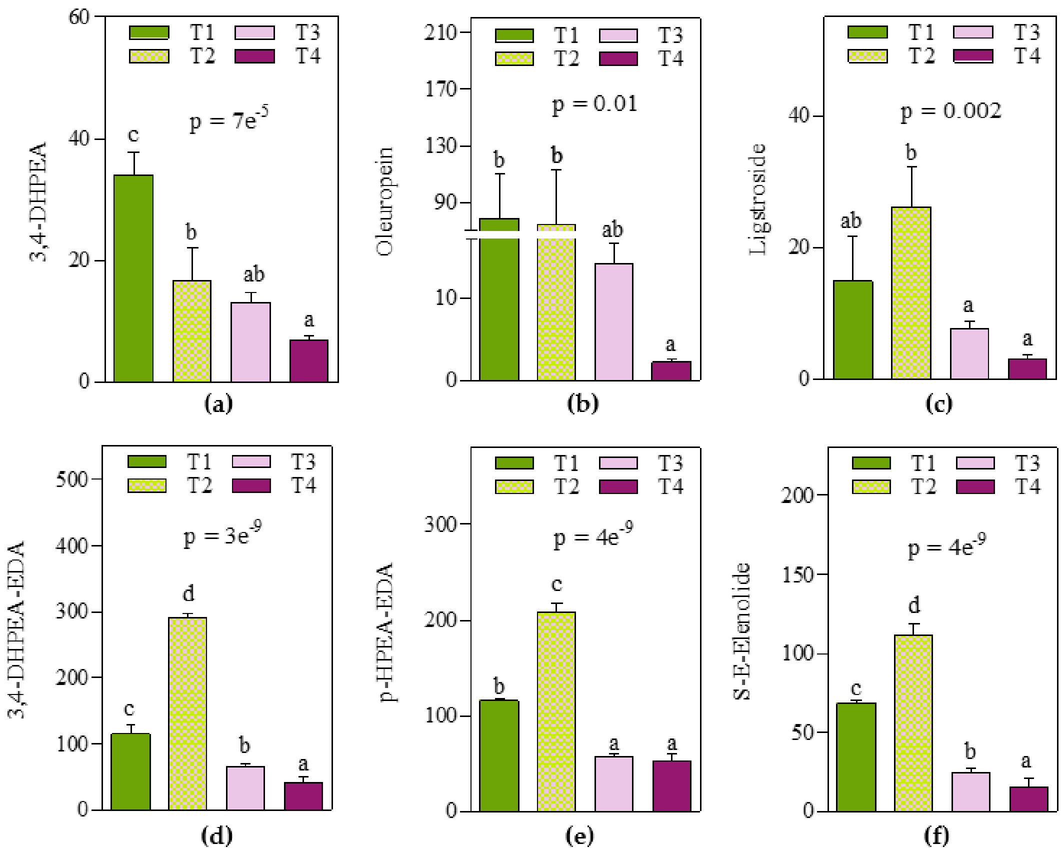

Figure S6. Mean values ± standard deviations of phenolic alcohols, secoiridoids and derivates (mg kg^{-1} FW): **(a)** 3,4-DHPEA (hydroxytyrosol); **(b)** Oleuropein; **(c)** Ligstroside; **(d)** 3,4-DHPEA-EDA (oleacein); **(e)** p-HPEA-EDA (oleocanthal); **(f)** S-(E)-Elenolide in fruits (**a, b, c**) or in oils (**d, e, f**) from olive ('cv Arbequina') trees subjected to T1, T2, T3 and T4. Data were analyzed by one-way ANOVA Tukey's post-hoc test at 0.05 probability level was applied and different letters indicated significant difference. P-values are reported in the graphs.

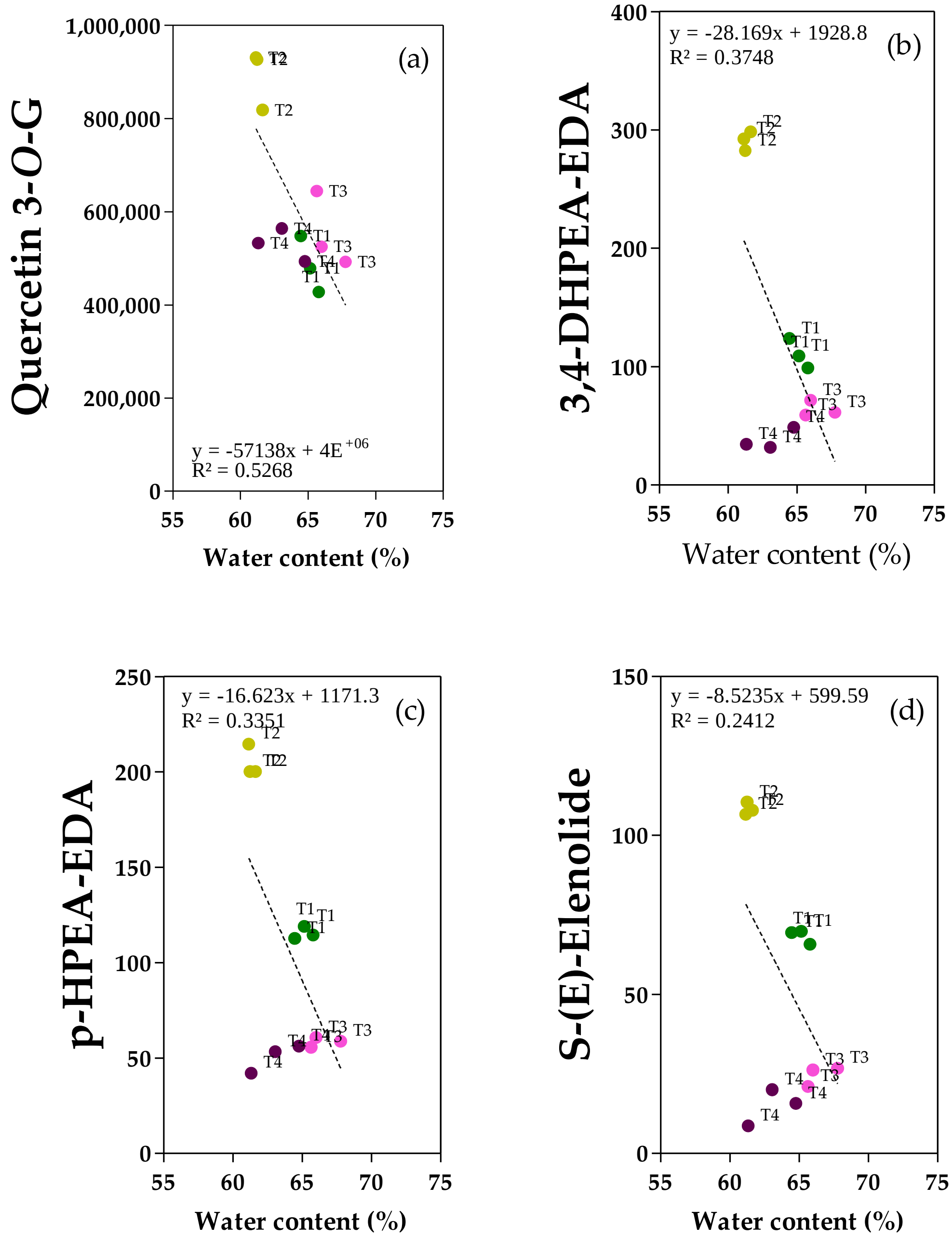

Figure S7. Regression analysis between water content (%) and (a) Quercetin-3-*O*-Glucoside (Quercetin-3-*O*-G) (ng g^{-1} FW) in fruits from olive ('cv Arbequina') trees subjected to T1, T2, T3 and T4; (b) 3,4-DHPEA-EDA, (c) p-HPEA-EDAand (d)S-(E)-Elenolide in oils (mg kg^{-1})from olive ('cv Arbequina') trees subjected to T1 (•) full irrigation with tilled soil, T2 (•) reduced irrigation with tilled soil, T3 (•) full irrigation with non-tilled soil and, T4 (•) reduced irrigation with non-tilled soil.

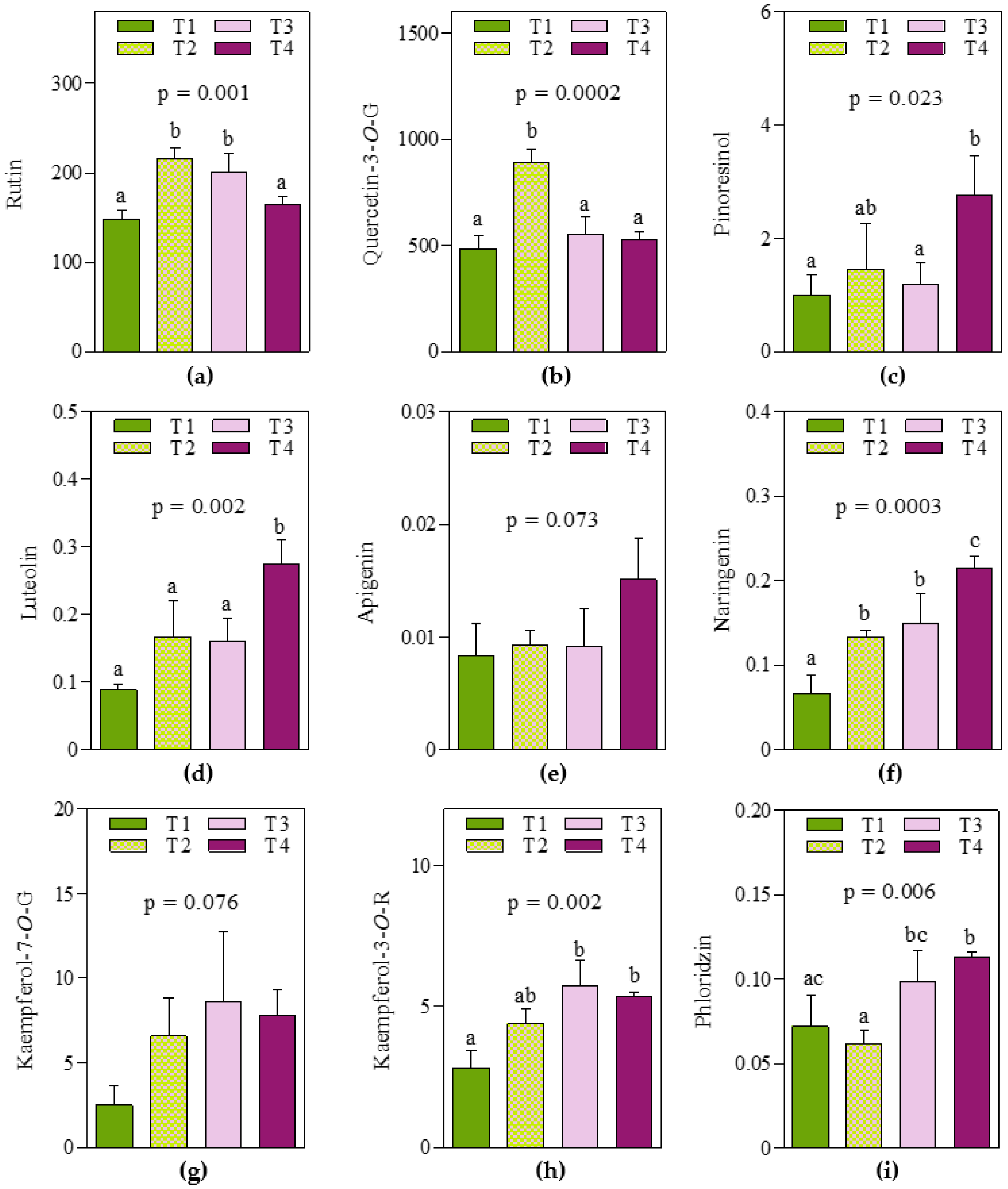

Figure S8. Mean values ± standard deviations of flavonoids and lignan (mg kg^{-1} FW): **(a)** Rutin; **(b)** Quercetin-3-O-Glucoside (Quercetin-3-O-G); **(c)** Pinoresinol; **(d)** Luteolin; **(e)** Apigenin; **(f)** Naringenin; **(g)** Kaempferol-7-O-Glucoside (Kaempferol-7-O-G); **(h)** Kaempferol-3-O-Rutinoside (Kaempferol-3-O-R); **(i)** Phloridzin in fruits from olive ('cv Arbequina') trees subjected to T1, T2, T3 and T4. Data were analyzed by one-way ANOVA Tukey's post-hoc test at 0.05 probability level was applied and different letters indicated significant difference. P-values are reported in the graphs.

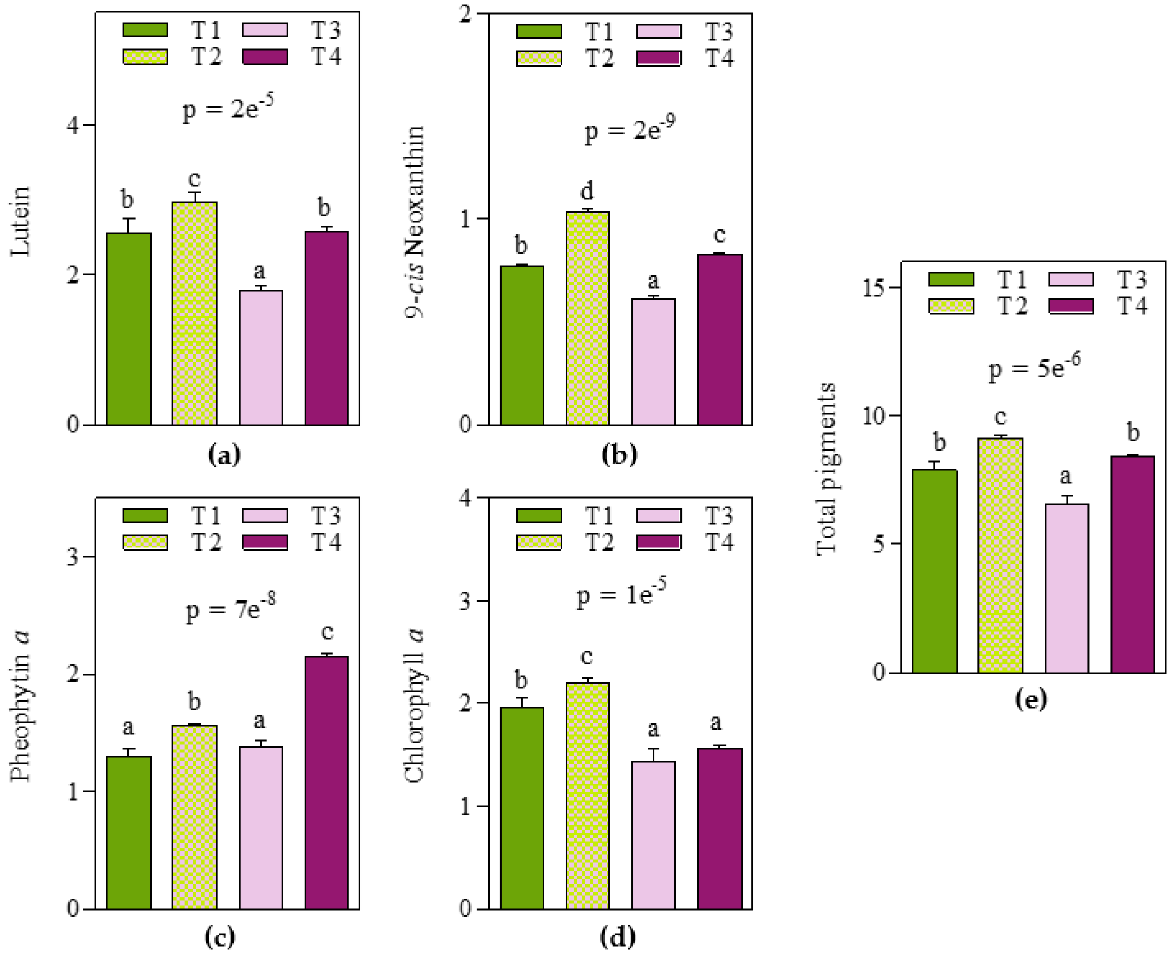

Figure S9. Mean values ± standard deviations of pigments (mg kg^{-1} oil): **(a)** Lutein; **(b)** *9-cis* Neoaxanthin; **(c)** Pheophytin *a*; **(d)** Chlorophyll *a*; **(e)** Total pigments in oils from olive ('cv Arbequina') trees subjected to T1, T2, T3 and T4. Data were analyzed by one-way ANOVA Tukey's post-hoc test at 0.05 probability level was applied and different letters indicated significant difference. P-values are reported in the graphs.

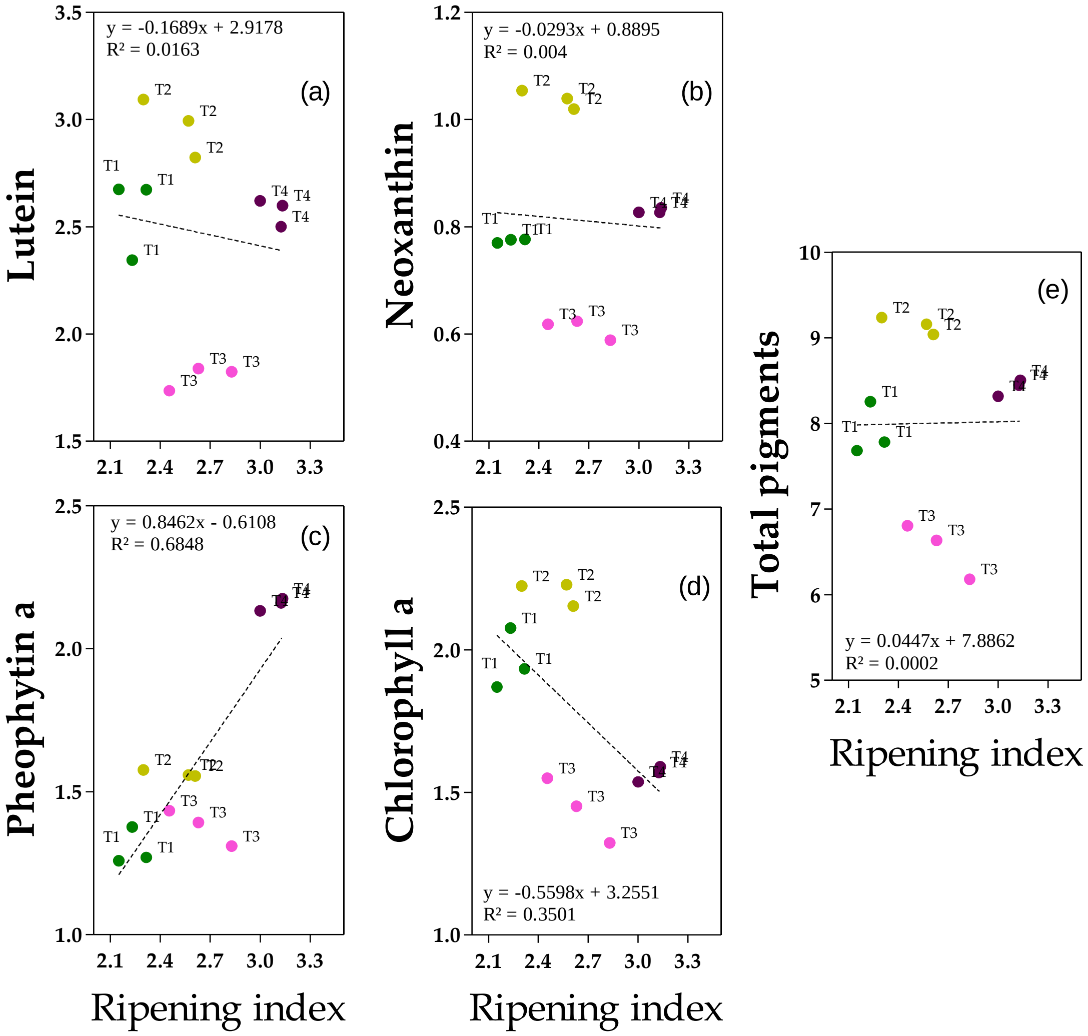

Figure S10. Regression analyses between ripening index and **(a)** lutein; **(b)** 9-cis neoxanthin (Neoxanthin); **(c)** pheophytin *a*; **(d)** chlorophyll *a*; **(e)** total pigments in oils (mg kg^{-1} oil) from olive ('cv Arbequina') trees subjected to T1 (•) full irrigation with tilled soil, T2 (•) reduced irrigation with tilled soil, T3 (•) full irrigation with non-tilled soil and, T4 (•) reduced irrigation with non-tilled soil.

Materials and methods

Common source parameters were as follows: nebulization gas (GS1) 50; turbo gas (GS2) 50; curtain gas (CUR) 10; temperature (TEM) 500 ◦C; ion-spray voltage (IS) -4500 V, input potential (EP) 10 V. The compound parameters, declustering potential (DP), collision energy (CE) and collision cell exit potential (CXP) were adjusted for the specific selected reaction monitoring transition (SRM) for each component. The SRM transitions and corresponding compound parameters are shown in Table S3.

Table S4. SRM transitions and corresponding phenols' parameters.

Compound Name	Q1	Q3	CE(eV)	CXP(V)	DP(V)
2-Coumaric Acid	163	119	-18	-11	-65
3-Coumaric Acid	163	119	-18	-11	-65
4-Coumaric Acid	163	119	-18	-11	-65
Vanillic acid	166.9	108	-26	-13	-62
Gallic acid	168.9	125	-20	-13	-75
Caffeic acid	178.9	135	-23	-11	-86
Cinnamic acid	147	103	-15	-16	-65
Protocatechuic acid	153	109	-21	-7	-86
trans-Ferulic Acid	193	134	-20	-8	-62
Cynarine	515.1	191	-41	-7	-120
Rosmarinic Acid	359	161	-22.5	-10	-70
Chlorogenic Acid	353	191	-24	-9	-61
Cichoric Acid	473.1	149	-30	-8	-76
Verbascoside	623.1	160.9	-43	-7	-82
Tyrosol	137	106	-22	-13	-78
Hydroxytyrosol	153	123	-20	-17	-100
Ligstroside	523.1	291	-32	-11.8	-117
Oleuropein	539.1	275	-32	-12	-137
Quercetin	301	150.9	-38	-8	-113
Quercetin 3-*O*-Glucoside	463.1	300	-37	-5	-154
Quecetin-3,4-*O*-DiGlucoside	625.1	270.9	-85	-12	-178
Rutin	609.2	299.9	-48	-11	-154
Quercetagetin 7-*O*-Glucoside	479.1	316.9	-31	-14	-152
Myricetin	316.9	150.9	-33	-9	-125
Kaempferol 3-*O*-rutinoside	593.2	284.9	-40	-5	-138
Kaempferol 7-*O*-glucoside	447.1	284.9	-38	-5	-158
Kaempferol 3-*O*-glucoside	447.1	284.1	-39	-11	-202
Tiliroside	593.201	284.901	-40	-5	-138
Naringenin	270.9	150.9	-25	-10.5	-120
Apigenin	268.9	117	-49	-14	-120
Luteolin	284.9	133	-44.6	-17.4	-130
Pinoresinol	357	151	-26	-7	-97
Catechin	289	244.9	-22	-11	-108
Epicatechin	289.001	244.901	-22	-11	-108
Procyanidin B1	577.1	289	-35	-11	-156
Procyanidin B2	577.1	289	-35	-11	-156
Procyanidin B3	577.1	289	-35	-11	-156
Piceid	389.1	227	-32	-11	-125
Resveratrol	227.1	185	-25	-9	-179
Phloretin	273	167	-38	-11	-103
Phloridzin	435.1	272.9	-23	-5	-135
